# Olfactory dysfunction in COVID-19: a marker of good prognosis?

**DOI:** 10.1016/j.bjorl.2020.12.002

**Published:** 2021-01-01

**Authors:** Cindy Vitalino Mendonça, José Arruda Mendes Neto, Fabio Akira Suzuki, Marlon Steffens Orth, Hugo Machado Neto, Sérgio Roberto Nacif

**Affiliations:** Hospital do Servidor Público Estadual Francisco Morato de Oliveira, Departamento de Otorrinolaringologia, São Paulo, SP, Brazil

**Keywords:** Olfaction disorders, Coronavirus infections, Dysgeusia, Smell

## Abstract

**Introduction:**

In May 2020, the World Health Organization recognized olfactory dysfunction as a COVID-19 symptom. The presence of hyposmia/anosmia may be a marker of good prognosis in COVID-19.

**Objective:**

To associate the presence of olfaction disorder to the clinical condition severity in patients with COVID-19.

**Methods:**

Individuals with the flu syndrome caused by SARS-CoV-2, diagnosed from March to June 2020, were recruited. They were divided into three groups: mild flu syndrome, severe flu syndrome (admitted to hospital wards) and critical illness (admitted to the ICU). Inpatients were interviewed by telephone contact after hospital discharge and their medical records were also evaluated regarding complementary test results. Outpatients answered an electronic questionnaire containing only clinical information.

**Results:**

A total of 261 patients participated in the study: 23.75% with mild flu syndrome, 57.85% with severe flu syndrome and 18.40% with critical illness. A total of 66.28% patients with COVID-19 had olfaction disorders. In approximately 56.58% of the individuals the smell alterations lasted between 9 days and 2 months. There was a significantly higher proportion of individuals with olfactory dysfunction in the group with mild flu syndrome than in the severe flu syndrome group (mild × severe – *p* < 0.001; Odds Ratio = 4.63; 95% CI [1.87–10.86]). This relationship was also maintained between patients with mild flu syndrome and critically-ill patients (mild × critical – *p* <  0.001; Odds Ratio = 9.28; 95% CI [3.52–25.53]).

**Conclusion:**

Olfaction dysfunction was significantly more prevalent in patients with mild flu syndrome in COVID-19. It may be a predictor of a good prognosis for this infection. New population-based studies must be carried out to corroborate these findings.

## Introduction

The continuous spread of the epidemic caused by the new coronavirus (SARS-CoV-2) has a massive impact on medical specialties and promotes a permanent search aiming to identify and understand possible new signs and symptoms of the new coronavirus 2019 disease (COVID-19). The most common clinical picture is characterized by fever, cough, dyspnea, myalgia and upper respiratory symptoms.^1^, ^2^

In May 2020, two months after it declared the COVID-19 pandemic, the World Health Organization (WHO) recognized alterations in the perception of smell and taste as symptoms of this disease.^3^ Records of such dysfunctions have reportedly been rare findings in Chinese studies, found in approximately 5% of cases.^4^ In a multicenter European study, 85.6% of the assessed patients reported alterations in smell and 11.8% had anosmia before the onset of other symptoms.^5^ In a study by the American Academy of Ototlaryngology/Head and Neck Surgery, 26.6% of individuals reported it as the only initial symptom.^6^ The Brazilian literature is still scarce on the subject. Jofilly et al. reported that the sudden loss of smell had a high positive predictive value (88%) for COVID-19 diagnosis during the epidemic in Brazil.^7^

Due to the clinical importance of this new disease and aiming to understand olfactory alterations related to this respiratory syndrome, this study was designed in a tertiary center in the city of São Paulo with patients diagnosed with COVID-19.

The aim of this study is to assess the association between olfactory disorders in patients with COVID-19 and the severity of the flu syndrome (mild flu syndrome, severe flu syndrome, critical illness).

## Methods

The study was carried out in a tertiary hospital and was approved by the local ethics committee CAAE 31512220.5.0000.5463. All participants signed an electronic free and informed consent form provided by the researchers. The data were collected between March and June 2020.

The inclusion criteria were patients over 18 years old with a COVID-19 diagnosis confirmed by reverse-transcriptase polymerase chain reaction (RT – PCR) of the nasopharynx and oropharynx swab or by serology.^1^, ^2^ Those with alterations in olfaction or taste prior to COVID-19 infection; hospitalized patients at the time of data collection for the study; tracheostomized patients; individuals with mental confusion after prolonged hospital stay and those who refused to sign the consent form were excluded.

The individuals were selected by non-probabilistic sampling by judgment. Three groups of patients were created for this study, according to the severity of the flu syndrome caused by SARS-CoV-2: severe flu syndrome (in-hospital treatment without the need for intensive support), critical illness (in-hospital treatment with the need for intensive support) and mild flu syndrome (outpatient treatment).

Inpatients (with and without olfactory dysfunction) were interviewed by a telephone call made by a team of doctors after hospital discharge, a period during which these patients did not have mental confusion and were able to answer the questionnaire prepared by the researchers. The questionnaire included identification data (age and gender) and the following clinical variables related to the period of SARS-CoV-2 infection: presence of nasal symptoms (nasal obstruction and/or nasal secretion), neurological symptoms (headache and /or dizziness), history of DM, smoking habits, alterations in olfaction and taste. In case of an affirmative answer for hyposmia/anosmia, they were also asked about the duration of this condition (<9 days, ≥9 days and <2 months, ≥2 months). There was no quantification through the visual analog scale of any of the symptoms. Laboratory parameters; C-reactive protein (CRP) and lymphocytes, were obtained from information recorded in the electronic medical record of individuals on the first day of hospital admission. Patients with mild flu syndrome (with and without olfactory dysfunction) were comprised of health professionals from this tertiary hospital. They were contacted and received an electronic questionnaire similar to that submitted to patients who were hospitalized, but laboratory parameters were excluded. These patients did not undergo test collection.

The sample size calculation for this cross-sectional study was based on the study by Mein ST et al. (2020),^8^ delineated in the Epi Info software, showing a 95% confidence level, 80% power and 5% margin of error. Using the Kelsey, Fleiss and Fleiss methods with continuity correction, the calculation of the total number of individuals was 210, 212 and 234, respectively.

### Statistical analysis

The variables considered for this study were: patient's gender, age, dysgeusia, diabetes mellitus, nasal symptoms, neurological symptoms, smoking status, clinical condition (mild flu syndrome, severe flu syndrome, critical illness), lymphocyte blood level and serum CRP level.

The programs SPSS V16, Excel office 2010 and EPI INFO 7.2.2.6 were used for the statistical analysis. A level of statistical significance <0.05 (5%) was used for this study.

Categorical variables were described according to frequency and the numerical variables according to mean and standard deviation (SD). Two-tailed Chi-Square test or two-tailed Fisher’s exact test was used to assess the difference between the frequencies of categorical variables.

Parametric and nonparametric tests were used to study the difference in means between the numerical variables, according to the Bartlett test result. When analyzing these variables, when the Bartlett test resulted in a value of *p* < 0.05, the test used was the Mann–Whitney/Wilcoxon (Kruskal–Wallis test). On the other hand, when the p-value in this test was >0.05, suggesting homogeneous variance, the ANOVA test (parametric) was used.

## Results

The sample consisted of 261 individuals. Sixty-two patients (23.75%) had mild flu syndrome, 151 (57.85%) had severe flu syndrome and 48 (18.40%) had critical illness.

Patients with mild flu syndrome were statistically younger (*p* < 0.001). They had significantly more nasal and neurological symptoms when compared to critically-ill (*p* < 0.001) and severe (*p* < 0.001) patients (Table 1).

**Table 1:** Clinical syndromes in COVID-19.

	Clinical syndromes in COVID-19			p-value		
	Mild Flu Syndrome (MFS)	Severe Flu Syndrome (SFS)	Critical Illness (CI)	MFS × SFS	MFS × CD	SFS × CD
	(n = 62)	(n = 151)	(n = 48)			
Age^a^	36.67 ± 10.25	63.18 ± 12.65	59.89 ± 12.89	<0.001^c^.^f^	<0.001^c^.^f^	0.12^c^
Gender (M/F)^b^	41.94%/58.06%	54.97%/45.03%	37.50%/62.50%	0.11^d^	0.78^d^	0.06^d^
Nasal symptom^b^	56.45%	19.87%	16.67%	<0.001^d^.^f^	<0.001^d^.^f^	0.67^d^
Neurological symptom^b^	70.97%	25.17%	29.17%	<0.001^d^.^f^	<0.001^d^.^f^	0.71^d^
DM^b^	4.84%	36.42%	45.83%	<0.001^d^.^f^	<0.001^d^.^f^	0.31^d^
Smoking status^b^	6.45%	4.64%	0.00%	0.73^e^	0.13^e^	0.19^e^
Total lymphocytes^a^	N/D	1177.95 ± 420.83	1251.16 ± 517.39	N/D	N/D	0.37^c^
CRP^a^	N/D	9.34 ± 8.02	14.69 ± 9.26	N/D	N/D	<0.001^c^.^e^

MFS, Mild Flu Syndrome; SFS, Severe Flu Syndrome; CI, Critical Illness; N/D, No data was collected.

a Values in means and standard deviation.

b Values in percentages.

c ANOVA test.

d Two-tailed corrected X^2^ test.

e Two-tailed Fisher's exact test.

f *p* < 0.05.

The prevalence of diabetes mellitus (DM) was statistically higher in hospitalized patients (*p* < 0.001) (Table 1). CRP levels were significantly higher in critically-ill patients than in patients with severe flu syndrome (*p* < 0.001) (Table 1).

Olfactory alteration was observed in 66.28% of patients with COVID-19. The duration of olfactory dysfunction was variable among the interviewees. Most patients had hyposmia/anosmia for more than nine days and less than two months (56.68%). A total of 51 patients (32.48%) had olfactory dysfunction that lasted less than 9 days and 17 patients (10.82%) had olfactory dysfunction that lasted more than 2 months. It is necessary to point out that 104 patients who had olfactory dysfunction were unable to accurately inform the duration of hyposmia/anosmia.

### Olfactory dysfunction and clinical picture severity

**Figure 1:**
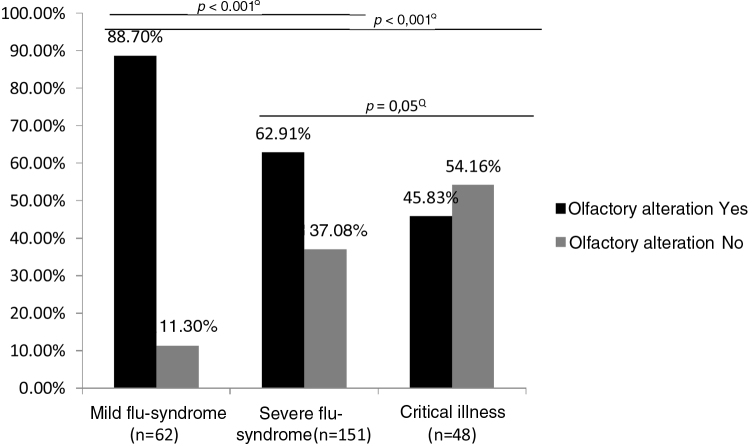
Distribution of olfactory alterations stratified by the clinical picture of COVID-19.

Olfactory dysfunction was observed in 88.70% of patients with mild symptoms; 62.91% of those admitted to the hospital and 45.83% of those admitted to the intensive care unit. There were significantly more patients with olfactory dysfunction among individuals with mild flu syndrome than among those with severe flu syndrome (*p* < 0.001; Odds Ratio = 4.63; 95%CI [1.87–10.86]). Moreover, there were significantly more patients with impaired olfaction among those with severe flu syndrome than in critically-ill patients (*p* = 0.05; Odds Ratio = 1.98; 95%CI [1.02–3.82]). Finally, this association was also maintained when comparing individuals with mild flu syndrome vs. critically-ill patients (*p* < 0.00); Odds Ratio = 9.28; 95%CI [3.52–25.53]) (Fig. 1).

### Olfactory dysfunction and dysgeusia

Patients with hyposmia/anosmia had significantly more dysgeusia than patients without this disorder (*p* < 0.001; Odds Ratio = 89.13; 95%CI [34.88–227.70]).

### Olfactory dysfunction and nasal symptoms

**Figure 2:**
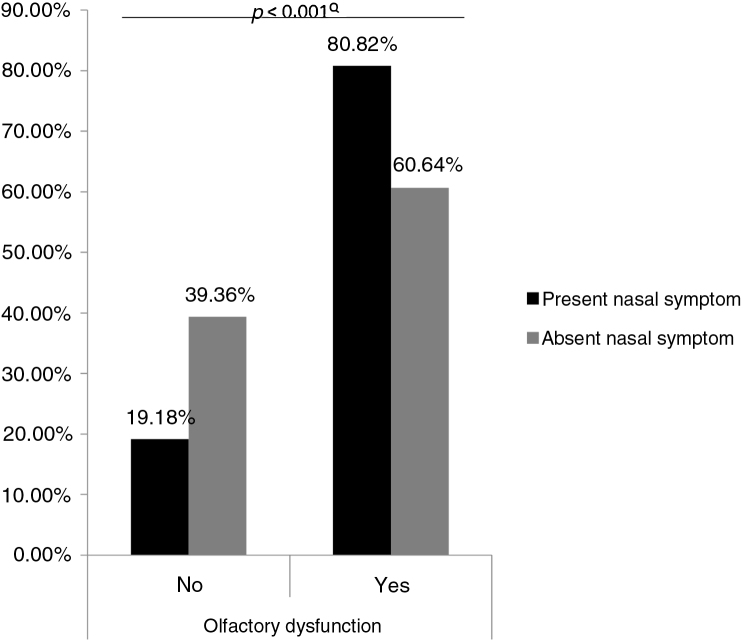
Olfactory dysfunction and nasal symptoms. Q, Two-tailed *X^2^* test.

When asked about the presence of nasal symptoms, 80.82% of the patients with olfactory dysfunction reported nasal obstruction and/or secretion. On the other hand, only 19.18% of patients without olfactory dysfunction reported rhinorrhea and/or nasal congestion (*p* < 0.001; Odds Ratio = 2.73; 95%CI [1.42–5.25]) (Fig. 2).

### Olfactory dysfunction and neurological symptoms

**Figure 3:**
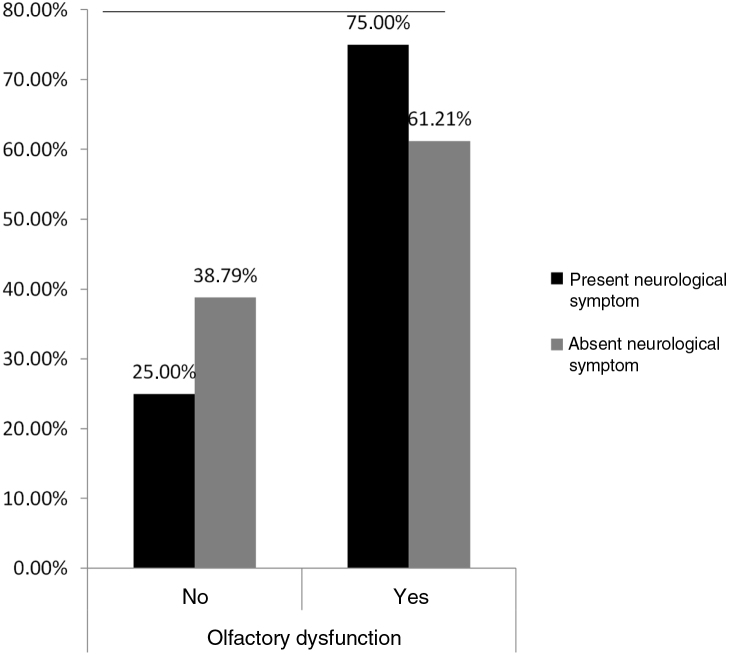
Olfactory dysfunction and neurological symptoms. Q, Two-tailed *X^2^* test.

There was a statistically significant association between the presence of olfactory disorders and neurological symptoms, such as dizziness and headache (*p* = 0.03; Odds Ratio = 1.90; 95%CI [1.08–3.32]) (Fig. 3).

## Discussion

Olfactory dysfunction is recognized by the World Health Organization (WHO) as one of the symptoms caused by SARS-CoV-2.^3^ Despite being widely observed in clinical practice and published in foreign studies,^9^, ^10^, ^11^ it is a disorder related to COVID-19 that has been minimally reported in the Brazilian literature.^7^, ^12^, ^13^ In the present study, we observed an inverse association between the presence of hyposmia/anosmia and the severity of the patient’s clinical condition.

Participants classified as having mild flu syndrome were statistically younger when compared to patients who required hospitalization. This result is similar to that found in other studies, in which older patients tend to have a more severe clinical picture of COVID-19, probably due to the higher number of chronic diseases and decreased immunological competence.^1^, ^2^

Patients in the mild flu syndrome group also had significantly more nasal and neurological symptoms. In a study carried out in South Korea with 213 outpatients, cough, hyposmia, dysgeusia, nasal obstruction, headache and myalgia were identified among the most common symptoms. In contrast, inpatients had more fever, cough and dyspnea.^14^

Diabetes mellitus was a significantly more frequent comorbidity in hospitalized patients. The presence of DM is considered a risk factor for developing the severe clinical form of COVID-19.^15^

Critically-ill patients had statistically higher levels of CRP. CRP level is considered an inflammatory marker and can be related to more severe conditions.^16^ Higher CRP values ​​may be associated with an increased need for care in the intensive care unit.^17^

In this study, 173 patients (66.28%) had some olfactory dysfunction during COVID-19 infection. In another study also carried out in Brazil, 79.2% of the studied patients had positive tests for COVID-19 and olfactory dysfunction (hyposmia and/or anosmia).^12^ This difference is probably due to the presence of only outpatients in the second study.

Most respondents in this study had a clinical complaint of hyposmia/anosmia lasting less than two months. Kosugi et al. (2020) followed the patients for an average of 31 days and verified that 52.6% of these participants achieved complete olfactory recovery in approximately 15 days.^12^

There was a significantly higher prevalence of hyposmia/anosmia in patients with the mild clinical condition when compared to patients with severe flu syndrome and those with critical illness. Moreover, there were statistically more cases of olfactory dysfunction among patients who were admitted to the hospital ward when compared to participants with a history of ICU admission. Similarly, Yan et al. (2020), evaluated 196 patients and concluded that those with anosmia or hyposmia had a lower rate of hospitalization when compared to those with normal olfactory function (OR = 0.09; 95%CI [0.01–0.74]).^18^ Therefore, they suggest that patients with flu syndrome caused by SARS-CoV-2, but without olfactory dysfunction should seek medical help earlier, because of the greater likelihood of pulmonary involvement and greater chance of developing the severe systemic inflammatory syndrome. Finally, they report that the presence of anosmia/hyposmia could be used as a clinical marker related to disease severity, just like the APGAR scale in the newborn.^18^ In contrast to these findings, Moein et al. (2020) and Vaira et al. (2020) found no association between the olfactory disorder and disease severity. Vaira stated that alterations in olfaction reported by critically-ill patients can be overlooked in the context of prolonged hospitalization and invasive ventilatory support.^8^, ^19^, ^20^ Another study carried out in the Brazilian population that included patients admitted to the ICU and the hospital ward and participants who received only outpatient treatment also did not identify an association between the presence of olfaction disorder and the severity of the patients’ clinical condition.^13^

There was also a significant association between the presence of hyposmia/anosmia and dysgeusia. Speth et al. (2020) found an important association between the clinical symptom of olfaction and taste alteration. They reported that the severity of taste dysfunction is closely associated to the severity of olfactory dysfunction.^21^ Although it is possible that SARS-CoV-2 acts on the chemoreceptors of smell and taste separately, these clinical complaints are more likely to be directly related due to impaired retronasal taste perception, which influences the experienced tastes.^21^

In the present study, patients with olfactory dysfunction reported significantly more nasal symptoms (nasal obstruction and / or secretion). In a study prior to the SARS-CoV-2 pandemic, another type of human coronavirus was inoculated into the upper airways of nineteen patients. Nine participants developed a common cold. It was observed that, in these patients, there was a hyperresponsiveness of the subepithelial microcirculation of the upper airways with increased plasma exudation, which resulted in mucosal edema and nasal obstruction.^22^ Based on this, it can be considered that, in SARS-CoV-2 infection, there may also be a conductive disorder that results in the mechanical blocking of odor molecules, which prevents them from reaching the chemoreceptors in the olfactory epithelium. This mechanism could also be responsible for generating olfaction disorders in patients with local symptoms.

Patients with olfactory dysfunction had a significantly higher frequency of neurological symptoms. One of the mechanisms proposed for the spread of SARS-CoV-2 to the central nervous system is through the cribriform plate, reaching the olfactory nerves and the olfactory bulb,^23^ leading to a picture of viral encephalitis. This pathophysiological mechanism could explain the presence of prolonged headache for a few weeks in some individuals with olfaction alterations.

The findings of this study suggest that patients with hyposmia/anosmia might have a state of better immunological competence, with a greater chance of containing SARS-CoV-2 in the upper airways. The olfactory dysfunction in individuals with COVID-19 could be caused either by edema in the nasal mucosa (conductive), or by viral aggression directly in the olfactory system (involvement of the olfactory epithelium and/or neurons). Another proposed mechanism for the development of post-viral hyposmia/anosmia would be the increase in cytokine production. Henkin et al. (2013) identified elevated levels of IL-6 in the nasal mucus and plasma of individuals with these symptoms after a cold, when compared to controls (29.7 vs. 11.6 pg/mL; *p* < 0.001).^24^ This interleukin could regulate the activity of the olfactory neurons and glial cells.^24^ In individuals with severe COVID-19, the serum levels of this substance are increased.^16^ On the other hand, in mild cases, there may be a localized increase of this mediator, thus promoting a localized inflammatory process and leading to a dysfunction in the olfactory system.

A limitation of this study is related to the inaccurate reporting of anosmia/hyposmia in hospitalized patients. Patients who were admitted to an intensive care unit or hospital ward may have had difficulties in reporting the presence of olfactory dysfunction during the acute picture of SARS-CoV-2 infection. Although the data regarding these patients were collected after hospital discharge, that is, during a period without mental confusion and without clinical alterations, the use of oxygen support during hospitalization (orotracheal intubation or use of a nasal catheter) and presence of more severe symptoms, such as dyspnea, can alter the patient's perception of the presence or absence of olfaction alterations. Another limitation of this study was the lack of quantification of olfaction loss. The results obtained, therefore, are restricted only to patients' reports. Moein et al. (2020) applied the validated UPSIT test (University of Pennsylvania smell identification test) to 60 inpatients with COVID-19 to measure olfactory dysfunction. About 98% of those studied had olfaction alterations, of which 83% had anosmia or severe hyposmia. A relevant information is that only 35% of this sample had olfactory dysfunction complaints before the test.^10^ Overall, olfaction alterations are more noticeable in cases of more significant loss, such as anosmia. Thus, although it was not quantified, it can be inferred that the number of olfactory dysfunctions may be higher than that recorded in individuals infected with SARS-CoV-2. Finally, despite the significant sample of patients, the present study was carried out in a single hospital, thus being geographically limited.

The olfactory dysfunction in COVID-19 seems to be more than a symptom of this new infection. The presence of hyposmia/anosmia in patients infected with SARS-CoV-2 may be a predictor of good disease prognosis. It is possibly a spectrum of this disease that affects mostly the upper airways, without significant pulmonary involvement. Therefore, it is reasonable to think that the presence of olfactory dysfunction as a symptom can be associated with a milder clinical picture of COVID-19.

## Conclusion

There was a significantly higher proportion of individuals with hyposmia/anosmia among patients with mild flu syndrome. Although the information about this dysfunction may be impaired by the clinical condition severity and by memory bias, the olfaction alteration has shown to be a factor of good prognosis for individuals infected with SARS-CoV-2. New population-based studies must be carried out to corroborate these findings.

## Conflicts of interest

The authors declare no conflicts of interest.
